# ECG changes after percutaneous edge‐to‐edge mitral valve repair

**DOI:** 10.1002/clc.23258

**Published:** 2019-09-09

**Authors:** Hou Bo, David Heinzmann, Christian Grasshoff, Peter Rosenberger, Christian Schlensak, Meinrad Gawaz, Jürgen Schreieck, Harald F. Langer, Johannes Patzelt, Peter Seizer

**Affiliations:** ^1^ Department of Cardiology and Angiology University Hospital, Eberhard Karls University Tübingen Tübingen Germany; ^2^ Department of Cardiology Affiliated Hospital of Qingdao University Qingdao Shandong Province China; ^3^ Department of Anaesthesiology University Hospital, Eberhard Karls University Tübingen Tübingen Germany; ^4^ Department of Cardiovascular Surgery University Hospital, Eberhard Karls University Tübingen Tübingen Germany; ^5^ Medical Clinic II, Universitäres Herzzentrum Lübeck, University Hospital Schleswig‐Holstein Germany; ^6^ German Center for Cardiovascular Research (DZHK), Partner Site Hamburg/Kiel/Lübeck Lübeck Germany

**Keywords:** atrial conduction, atrial strain, electrocardiogram, mitral regurgitation, percutaneous mitral valve repair, remodeling

## Abstract

**Background:**

Mitral regurgitation (MR) has a severe impact on hemodynamics and induces severe structural changes in the left atrium. Atrial remodeling is known to alter excitability and conduction in the atrium facilitating atrial fibrillation and atrial flutter. PMVR is a feasible and highly effective procedure to reduce MR in high‐risk patients, which are likely to suffer from atrial rhythm disturbances. So far, electroanatomical changes after PMVR have not been studied.

**Hypothesis:**

In the current study, we investigated changes in surface electrocardiograms (ECGs) of patients undergoing PMVR for reduction of MR.

**Methods:**

We evaluated 104 surface ECGs from patients in sinus rhythm undergoing PMVR. P wave duration, P wave amplitude, PR interval, QRS duration, QRS axis, and QT interval were evaluated before and after PMVR and at follow‐up.

**Results:**

We found no changes in QRS duration, QRS axis, and QT interval after successful PMVR. However, P wave duration, amplitude, and PR interval were significantly decreased after reduction of MR through PMVR (*P* < .05, respectively).

**Conclusion:**

The data we provide offers insight into changes in atrial conduction after reduction of MR using PMVR in patients with sinus rhythm.

## 1 INTRODUCTION

Mitral valve regurgitation (MR) contributes significantly to morbidity and mortality of heart failure and is the second most frequent indication for valvular repair.^1^ With advances in catheter‐based mitral valve repair using an edge‐to‐edge repair strategy (PMVR), a nonsurgical treatment option has successfully been established in clinical practice (MitraClip). While a reduction of MR has obvious hemodynamical advantages by increasing the effective left ventricular stroke volume and cardiac output,^2^ other secondary effects have been described. In a previous study, we have found that improved mitral valve coaptation and improved mitral valve annular size correlates with residual MR after PMVR using the MitraClip system.^3^ Furthermore, a recent study has found a significant decrease in left atrial operating chamber stiffness after deployment of PMVR.^4^ Increased strain of the left atrium through MR induces a significant remodeling of the affected atrial myocardium, which is an ideal substrate for atrial rhythm disturbances, especially atrial fibrillation (AF).^5^ With a reduction of MR, a reverse remodeling of the left atrium could be beneficial to preserve normal electrical conduction and hemodynamical function.

In the current study, we were therefore interested whether a change in hemodynamics through PMVR alters electrical activation of the atria. Thus, we performed an extensive analysis of patients undergoing PMVR to establish whether atrial unloading induces changes in electrocardiogram (ECG) morphology.

## 2 METHODS

### 2.1 Study cohort

**Figure 1:**
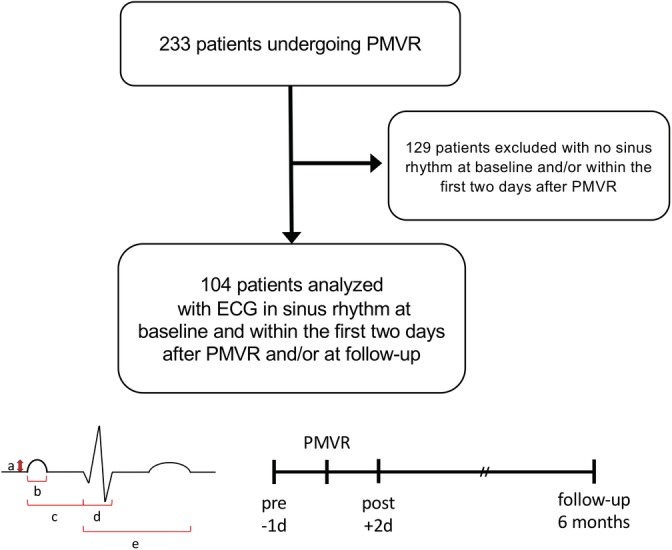
Flow chart of patient cohort and evaluation strategy. Of 233 patients undergoing percutaneous mitral valve repair (PMVR), 104 patients were included in the study with electrocardiogram (ECG) in sinus rhythm at baseline and within the first 2 days after PMVR and/or at the follow‐up visit to evaluate P wave characteristics (a). Patients with a previous medical history of atrial fibrillation (AF) were included in the analysis when ECGs at the specified timepoints showed sinus rhythm. (b) illustrates obtained ECG measurements including P wave amplitude (a), P wave duration (b), PR interval (c), QRS duration (d), and QT interval (e) within the time course of PMVR

Of 233 patients undergoing PMVR using the MitraClip system (Abbott Vascular, Chicago, Illinois) with MR grade 2+ to 4, patients were included in the study with ECG in sinus rhythm at baseline and within the first 2 days after PMVR and/or at the follow‐up visit to evaluate P wave characteristics (n = 104, Figure 1a). Patients with a previous medical history of AF were included in the analysis when ECGs at the specified timepoints showed sinus rhythm. The mean follow‐up time was 6.95 months. Interdisciplinary decision to perform PMVR was made based on the EuroSCORE (European System for Cardiac Operative Risk Evaluation) and additional relevant risk factors for poor surgical outcome. Adequate pharmacological heart failure treatment, according to current guidelines, had to be established at least 3 months prior the intervention. Of the patients in the cohort on anticoagulation, 48% took NOACs, whereas 41% of patients took vitamin K antagonists. Patients meeting predefined exclusion criteria^6^ were not treated with PMVR. The study was approved by the local ethics committee (#260/2015R).

### 2.2 Electrocardiogram analyses

Standard 12‐lead ECGs with a paper speed of 50 mm/s were obtained before and within 2 days after PMVR, as well as at follow‐up. A manual analysis of all ECGs was performed by an investigator blinded to the PMVR results, evaluating mean P wave duration, P wave amplitude, PR interval, QRS duration, and QT interval in three consecutive complexes in lead II. For analyses of P wave duration and amplitude, no fusion with a QRS complex or T wave was allowed and a stable isoelectric line had to be present. P wave duration was measured between the junctions of the P wave with the isoelectric line; P wave amplitude was measured from the peak/nadir of the P wave to the isoelectric line. Length of PR interval was determined from the junction of the P wave with the isoelectric line to the onset of the QRS complex. Length of the QRS interval was measured from its earliest deflection from the isoelectric line to its offset. Length of the QT interval was measured from the beginning of the QRS complex to the end of the following T wave. (Figure 1b) QRS axis was obtained by automated analysis of extremity leads. Evaluation of all ECGs was performed by an experienced investigator, blinded to clinical parameters.

### 2.3 Echocardiographic assessment

MR severity, left ventricular function, and left atrial diameter (LA diameter) were evaluated by standard evaluation methods, using Philips CX 50 or iE 33 cardiovascular ultrasound machines with transthoracic and transesophageal probes (Philips HealthCare, Hamburg, Germany). Severity of MR was determined according to the guidelines of the European Association of Echocardiography at baseline and at follow‐up.^7^ MR severity after the intervention was assessed using a method described by Foster et al.^8^

### 2.4 Statistical analysis

Statistical analysis was performed using GraphPad Prism (Ver. 8, GraphPad Software, La Jolla, California) and SPSS (Ver. 24, IBM Deutschland GmbH, Ehningen, Germany). Categorical variables are expressed as absolute numbers or as percentage, continuous variables as mean ± SEM, or mean ± SD were specified. Paired *t* test was used to compare means, and *P* < .05 was considered statistically significant.

## 3 RESULTS

### 3.1 Baseline characteristics

**Figure 2:**
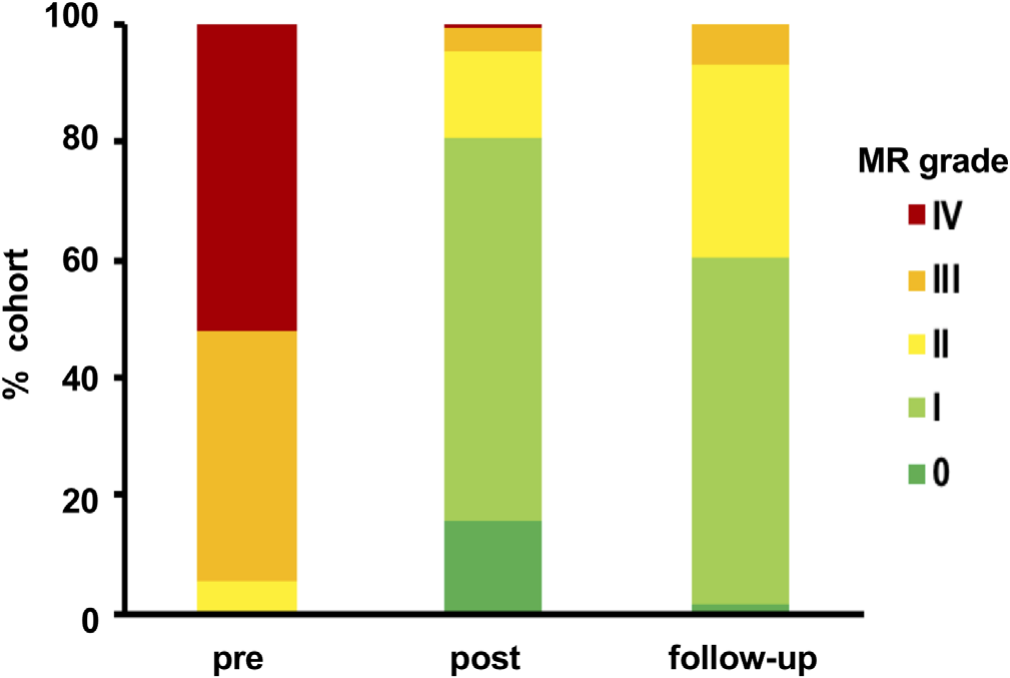
Reduction of mitral regurgitation (MR) using percutaneous mitral valve repair (PMVR). MR grades of patients undergoing PMVR are illustrated pre‐ and post‐PMVR, as well as at follow‐up. Grading was performed according to the guidelines of the European Association of Echocardiography

The baseline characteristics of the patient cohort are summarized in Table 1. The reduction of MR after PMVR is illustrated in Figure 2.

**Table 1:** Baseline characteristics

Variable	Study cohort (n = 104)	All screened patients (n = 233)
Age	72.8 ± 10.8 (104)	75.8 ± 9.3 (233)
Female gender	43.3% (45/104)	49.8% (117/233)
LV function		
≤ 35%	52.0% (54/104)	50.2 (117/233)
36‐50%	24.0% (25/104)	25.3 (59/233)
> 50%	24.0% (25/104)	24.5% (57/233)
NYHA class pre	3.2 ± 0.6 (101)	3.1 ± 0.6 (228)
NYHA class FU	2.1 ± 0.6 (65)	2.3 ± 1.2 (151)
EuroSCORE II	8.6 ± 6.9 (104)	9.7 ± 8.8 (232)
Chronic renal failure	51.0% (53/104)	51.5% (120/233)
Coronary artery disease	81.7% (85/104)	75.5% (176/233)
Hypertension	69.2% (72/104)	73.4% (171/233)
Pulmonary hypertension	57.3% (59/103)	61.9% (143/231)
Diabetes mellitus	29.8% (31/104)	29.2% (68/233)
Insulin dependent diabetes	12.5% (13/104)	10.7 (25/233)
Previous cardiac surgery	28.8% (30/104)	30.0% (70/233)
Chronic lung disease	7.7% (8/104)	6.0% (14/233)
Recent myocardial infarction	14.4% (15/104)	12.4% (29/233)
Extracardiac arteriopathy	24.0% (25/104)	22.7% (53/233)
Hyperlipoproteinemia	47.1% (49/104)	47.6% (111/233)
MR preintervention (grades)	3.5 ± 0.6 (104)	3.5 ± 0.5 (233)
MR postintervention (grades)	1.3 ± 0.7 (103)	1.3 ± 0.7 (232)
MR at follow‐up (grades)	1.7 ± 0.6 (71)	1.7 ± 0.6 (161)
MR genesis functional	45.1% (46/102)	46.9% (107/228)
6 min walk test preintervention (m)	158 ± 124	145 ± 114 (142)
6 min walk test at follow‐up (m)	260 ± 133	265 ± 135 (127)
Average number of rehospitalizations for heart failure within 6 mo post intervention	0.21 ± 0.48 (66)	0.25 ± 0.53 (153)
Atrial fibrillation	35.6% (37/104)	65.2% (152/233)
LA diameter	38.7 ± 9.3 (87)	41.3 ± 10.4 (200)
Heart rate preintervention	71 ± 15 (69)	70 ± 16 (156)
Heart rate postintervention	70 ± 21 (60)	71 ± 19 (145)
Betablockers	89.1% (90/101)	90.0% (207/230)
ACE/AT1‐inhibitors	82.2% (83/101)	81.1% (185/228)
Aldosterone antagonist	51.5% (52/101)	50.9% (116/228)
Diuretics	91.1% (92/101)	88.6% (203/229)
Digitalis	2.0% (2/101)	9.6% (22/228)
Calcium antagonist	15.3% (15/98)	18.3% (41/224)
Anticoagulation	45.1% (46/102)	68.3% (157/230)

Variables are expressed as mean ± SD.

Abbreviations: bpm, beats per minute; FU, follow‐up; LV, left ventricular; MR, mitral regurgitation; NYHA: New York Heart Association; y, years.

### 3.2 P wave duration and P wave amplitude are decreased after PMVR

**Figure 3:**
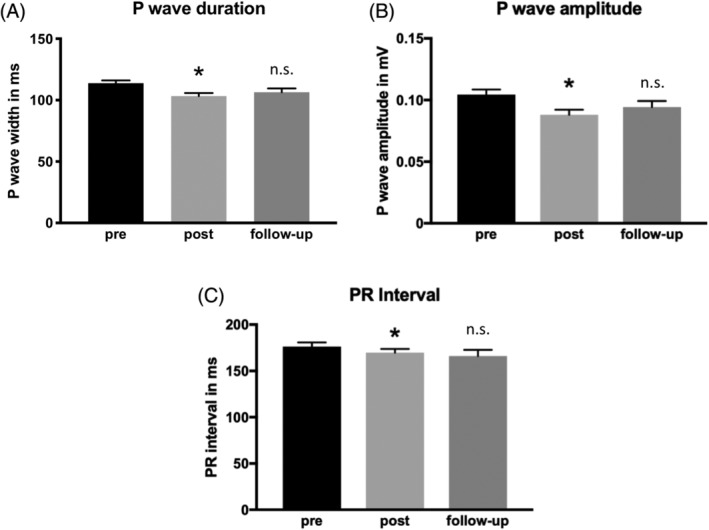
Reduction of mitral regurgitation using percutaneous mitral valve repair induces changes in atrial conduction. Surface ECGs of 104 patients with severe mitral regurgitation (MR) undergoing percutaneous mitral valve repair (PMVR) were analyzed. P wave duration in ms (a), P wave amplitude in mV (b), and PR interval in ms (c) are illustrated as mean ± SEM. All three parameters showed a significant decrease after reduction of MR. Parameters did not show significant changes during follow‐up. *indicates *P* < .05 compared to baseline; n.s. indicates *P* > .05 compared to post‐PMVR

At baseline, patients undergoing PMVR showed a mean P wave duration of 113.8 ± 2 ms before the intervention. In the ECG control shortly after the intervention, a reduction of the P wave duration to 103.3 ± 2 could be observed (*P* < .0001). At follow‐up, no significant change compared to post‐PMVR was apparent (106.4 ± 3 ms, *P* > .05 compared to post‐PMVR), as shown in Figure 3. Similar to the shortening of the P wave duration, patients undergoing PMVR showed a decrease of the P wave amplitude compared to the preinterventional results (0.104 ± 0.004 mV vs 0.088 ± 0.004 mV, *P* < .001). At follow‐up, no significant change of amplitude was found (0.094 ± 0.005 mV, *P* > 0.05 compared to post‐PMVR), as illustrated in Figure 3.

### 3.3 PR interval shortens after PMVR

Furthermore, we observed a decrease in PR interval from 176.3 ± 4.5 ms to 169.8 ± 4 ms after PMVR (*P* < .05). Similar to the other atrial parameters, the mean PR interval did not change significantly during follow‐up compared to post‐PMVR (166 ± 6.7 ms, *P* > .05, Figure 3).

### 3.4 Ventricular excitation is unchanged by PMVR

**Figure 4:**
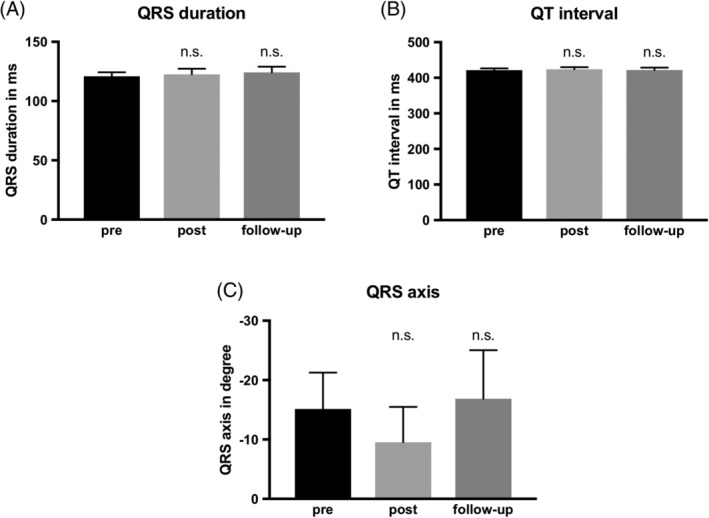
Ventricular excitation in surface electrocardiogram (ECG) does not change after PMVR. A total of 104 patients undergoing percutaneous mitral valve repair (PMVR) were analyzed regarding changes in QRS duration in ms (a), QT interval in ms (b), and QRS axis in degree (c). Reduction of mitral regurgitation using PMVR showed no significant change in ventricular excitation, neither shortly after the procedure nor at follow‐up. All parameters are shown as mean ± SEM, n.s. indicates *P* > .05

PMVR had no effect on QRS duration, neither after the procedure, nor did it induce changes at follow‐up (120.9 ± 3.4 ms vs 122.5 ± 4.8 ms [*P* > .05] vs 124.2 ± 4.8 ms [*P* > .05], Figure 4).

The QT interval was unchanged after the procedure and did not change during the follow‐up period (421.3 ± 5.1 ms vs 424.1 ± 5.6 ms [*P* > .05] vs 421.9 ± 6.9 ms [*P* > .05]).

Furthermore, we found no shift in the QRS axis after PMVR and, similarly to the aforementioned ventricular parameters illustrated in Figure 4, during the follow‐up period (−15.2° ± 6.1 vs ‐9.5° ± 6 [*P* > .05] vs ‐16.9° ± 8 [*P* > 0.05]).

## 4 DISCUSSION

PMVR is now a widely used approach to reduce MR in patients unsuitable for surgical repair of the mitral valve. With MR being a well‐established risk factor for severe left atrial remodeling, PMVR might be a tool to reduce atrial strain and have a beneficial effect on atrial conduction.

In this study, we describe for the first time distinct changes of atrial conduction found in surface ECGs of patients undergoing PMVR: (a) after PMVR, P wave duration PR interval, and P wave amplitude showed a strong decrease compared to baseline, (b) at follow‐up, P wave duration, PR interval, and P wave amplitude remained unchanged compared to post‐PMVR values, (c) QRS duration, QRS axis, and QT interval were not affected by PMVR.

In our cohort, patients undergoing PMVR experienced a significant reduction of MR, illustrated in Figure 2.

Atrial enlargement has been found to be an independent predictor for onset of AF after mitral valve repair, with atrial remodeling being the major culprit of electrical instability and susceptibility for AF.^9^ We and others have observed that PMVR results in significant geometrical changes, especially of the left atrium.^3^, ^10^ On the cellular level, Foglieni et al. have shown enhanced cardiomyocyte hypertrophy and higher intracytoplasm cytochrome c levels, as well as increased myocardial fibrosis in atria of patients with severe MR, illustrating the substrate changes that occur with prolonged atrial strain.^5^ With increased mechanical strain of the left atrium, several mechanisms have been elucidated that increase susceptibility to supraventricular rhythm disturbances. Ravelli and Allessie published findings in Langendorff‐perfused rabbit hearts, where an experimental increase in atrial pressure resulted in an increased susceptibility to AF that correlated with a shortening of atrial effective refractory periods (AERP).^11^ In line with our findings, Soylu et al. have reported an increase of AERP and their dispersion after percutaneous mitral balloon commissurotomy in patients with mitral stenosis.^12^ Although no follow‐up data were presented, the authors found convincing evidence that acute relief of chronic strain results in distinct improvement of atrial conduction. Weinsaft et al. found that patients with severe MR and LA dilation show an increased P wave amplitude and duration.^13^ Furthermore, Kamphuis et al. found a significant decrease of P wave amplitude directly after percutaneous closure of atrial septum defects, resulting in an acute reduction of atrial strain.^14^

With a reduction of MR through PMVR, a similar effect could be observed in our patient cohort, resulting in a reduction of P wave amplitude and duration immediately after the procedure. As the shortening of P wave duration and P wave amplitude, as well as the decrease in PR interval was apparent immediately after the reduction of MR, changes in atrial strain resulting in cellular excitability could be the primary underlying mechanism. Several ion channels have been found to be susceptible to atrial stretch in a fashion that alters susceptibility to atrial rhythm disturbances.^15^, ^16^, ^17^ Especially in terms of AF, this might be of significance, as Doi et al. have recently found a correlation between an increased P wave duration and higher recurrence rates in patients with AF.^18^ Similar results have been published based on cohorts of the Framingham Heart Study and Atherosclerosis Risk in Communities Study participants for increased P wave duration and PR interval.^19^ Furthermore, a decrease of P wave amplitude is associated with lower recurrence rates after cryoballoon ablation of AF.^20^ It is therefore stimulating to speculate whether the observed changes in atrial conduction and excitation have a clinically relevant impact on the occurrence of atrial rhythm disturbances, especially AF. The combination of reverse atrial remodeling regarding electromechanical and geometrical changes and reduced atrial strain through a reduction of regurgitation could stabilize the atrial substrate, reducing new onset of AF.

We did not see changes of atrial conduction during the follow‐up period, which could be due to the fact that PMVR is usually performed in patients with a protracted history of myocardial and cardiovascular disease promoting atrial remodeling. Furthermore, the follow‐up period might have been too short to see long‐term effects of atrial reverse remodeling after initial unloading of the left atrium has equilibrated. Interestingly, we did not observe significant changes in ventricular conduction, although PMVR increases the effective left ventricular stroke volume through reduction of MR.

## 5 LIMITATIONS

This study retrospectively evaluated 104 patients undergoing PMVR due to severe MR regarding changes in atrial conduction. Out of 233 patients undergoing PMVR, 104 patients were in sinus rhythm upon ECG evaluation, which was necessary to determine atrial parameters. The mean follow‐up duration of 6.95 months is a possible limitation of the study, as long‐term remodeling effects on the one hand and gradual increase of MR after PMVR on the other could affect outcome and secondary comorbidities, especially AF. Although there is strong evidence that prolonged P wave duration over 120 ms results in a higher risk of AF recurrence,^21^ the clinical implications of the demonstrated reduction in P wave duration from 113 to 106 ms in combination with reduced MR and subsequently reduced strain of the left atrium remain to be elucidated.

### CONCLUSION

In the current study, we provide data illustrating a decrease of P wave duration and amplitude, as well as a reduction of PR interval through reduction of MR using PMVR.
